# Symptom clusters and impact on quality of life in esophageal cancer patients

**DOI:** 10.1186/s12955-022-02084-9

**Published:** 2022-12-23

**Authors:** Yanli Wang, Zhongfei Xie, Yuxia Liu, Jianing Wang, Zhijun Liu, Sihan Li

**Affiliations:** Department of Radiation Oncology, The First Hospital of China Medical University, Shenyang, Liaoning Province China

**Keywords:** Esophageal cancer, Symptoms clusters, Social support, Quality of life

## Abstract

**Background:**

Esophageal cancer patients can experience co-occurring, related symptoms labeled symptom clusters. This study aimed to identify symptom clusters and explore which SCs independently affect the quality of life (QoL) among esophageal cancer patients.

**Methods:**

This cross-sectional study was performed in Shenyang, China, from February 2021 to February 2022. Finally, 118 esophageal cancer patients effectively completed the survey. Questionnaires’ information included the Functional Assessment of Cancer Therapy-General (FACT-G), the MD Anderson Symptom Inventory Gastrointestinal Cancer Module (MDASI-GI), the Multidimensions Scale of Perceived Social Support, and demographic and clinical characteristics. Exploratory factor analysis with principal axis factoring was used to identify symptom clusters, and multiple regression analysis was employed to analyze the influencing factors of QoL.

**Results:**

The mean score of FACT-G was 69.88 (SD = 17.85) among 118 esophageal cancer patients. Four symptom clusters were identified: psychological-somatic, dysphagia, fatigue-pain, and gastrointestinal symptom clusters. Results of regression analysis indicated a significant impact on QoL for chemotherapy (*β* = 0.140, *P* < 0.045), psychological-somatic symptom cluster (*β *= − 0.329, *P* = 0.013), and social support (*β* = 0.409, *P* < 0.001) after adjusting demographic and clinical characteristics. The linear combination explained 47.8% of the variance in QoL.

**Conclusions:**

There is a critical need to emphasize the importance of psychological-somatic symptoms clusters management programs and increasing social support to improve QoL in esophageal cancer patients.

## Introduction

Esophageal cancer is among the most prevalent malignant tumors in the upper digestive tract. Based on CLOBOCAN 2020 [1], esophageal cancer is the seventh most commonly diagnosed cancer and the sixth leading cause of cancer death, with an estimated 604,000 new cases and 544,000 deaths worldwide. In China, it was estimated in 2020 that the age-standardized (world) incidence and mortality of esophageal cancer were 13.80 and 12.70 per 100,000 in China, respectively. It has been ranked third as a leading cause of cancer death [1]. Esophageal cancer is characterized by increasing incidence, demanding treatment, and poor prognosis, with a five-year survival, is about 20% in China [2]. Although prognosis improves owing to early detection and treatment, esophageal cancer patients still undertake significant physical and psychological burdens [3].

Evidence found that cancer patients reported an average of 8 to 13.5 symptoms, depending on the cancer type and care level [4, 5]. Most patients with esophageal cancer seek medical attention owing to dysphagia. Though dysphagia is just a symptom of esophageal cancer, it causes adverse effects on health due to difficulty swallowing or weight loss [6]. Besides, esophageal cancer patients are susceptible to experiencing other symptoms, such as pain, fatigue, vomiting, dry mouth, and taste changes. Emerging research has shed light on symptom clusters (SCs) assessment, which may have a prognostic value in cancer patients [7]. Co-occurring and interrelated symptoms can be defined as symptom clusters [8, 9], and those in one group are distinct from other SCs and have different symptoms [10, 11]. Though some studies have explored the possibilities of SCs in esophageal cancer patients, the results were inconsistent [12, 13]. Furthermore, SCs have a more significant effect on QoL due to synergistic action than each symptom alone [14]. Therefore, the classification of SCs needs further exploration. Besides, the associations between SCs and QoL have not been widely studied in esophageal cancer patients.

Social support refers to the perception or actuality of being loved, cared for, respected, and valued in times of need by family members, friends, colleagues, and other communities in general [15]. According to the “strain buffering hypothesis” [16], social support can act as a “direct agent” to affect individuals’ well-being and health. Besides, social support, as an “antecedent factor”, positively affects mental health by strengthening psychological adjustment. Social support has been reported to significantly and positively affect QoL among cancer patients [17–19]. Individuals’ social relationship networks can be changed because of cancer diagnosis, which may affect the feeling of perceiving assistance available from social relationship networks during illness [17]. For esophageal cancer patients, sharing a meal with family or friends is a social event because difficulty swallowing or food regurgitation may result in withdrawal from social situations and isolation [6]. Hence, we supposed that social support was an independent factor affecting QoL in esophageal cancer patients.

Therefore, the primary purpose of this study was to investigate how symptoms clustered, and the secondary objective was to explore the effects of SCs and social support on QoL in esophageal cancer patients.

## Methods

### Study design and sample

This study was conducted in cross-sectional design in Shenyang, Liaoning province, China, from February 2021 to February 2022. Participants were recruited at the first affiliated hospital of China Medical University. The inclusion criteria of this study were that patient (1) was diagnosed with esophageal cancer; (2) could understand and communicate with Chinese; (3) was at least 18 years old. The exclusion criteria of this study included those patients who (1) had other severe diseases (such as severe cardiovascular disease; (2) had a history of psychiatry, cognitive and intellectual disorders. Each eligible patient would be distributed self-reported questionnaires after writing the study’s informed consent. The study was approved by the Ethics Committee on Human Experimentation of the First affiliated hospital of China Medical University (NO. 2021-430-2), and the study procedures followed ethical standards.

### Demographic and clinical information

Besides, several demographics and clinical information were included in the present study, including age, gender, marital status, educational background, residence, monthly income, time since diagnosis, chronic diseases condition, and whether to receive chemotherapy or surgery.

### Quality of life (QoL)

The Chinese version of the Functional Assessment of Cancer Therapy-General (FACT-G) [20] was used to assess the quality of life among esophageal cancer patients. The scale contains 27 items to measure cancer patients’ physical well-being (PWB), emotional well-being (EWB), social/family well-being (S/FWB), and functional well-being (FWB) [21]. Each item was scored on a five-point scale from 0 (not at all) to 4 (extremely), and a higher score reflects better QoL.

### Cancer-related symptoms

The present study assessed symptoms using a 13-item core symptoms subscale and a five-item gastrointestinal cancer-specific subscale from the MD Anderson Symptom Inventory Gastrointestinal Cancer Module (MDASI-GI) [22]. The Chinese version of MDASI-GI has been translated and validated by Chen et al [23]. It consists of 18 symptoms, and the severity of each symptom is scored on an 11- point Likert type (0= “not at all” to 10= “as bad as you can imagine”). A higher score reports more severe physical symptoms among esophageal cancer patients. According to the cut-off value, Bacorro et al [24] defined 0 as “no symptoms”, 1–3 as “mild symptoms”, 4–7 as “moderate symptoms”, and 8–10 as “severe symptoms”. Besides, non-zero scores mean the occurrence of symptoms.

### Social support

The Chinese version of the Multidimensions Scale of Perceived Social Support (MSPSS) [25] was used to test the level of social support in patients with esophageal cancer. The scale includes 12 items that are scored on a 7-point Likert type (from 1= “very strongly disagree” to 7= “very strongly agree”) and has three dimensions, including family support, friends support, and other necessary relationship support. The total score is from 12 to 84, with a higher score suggesting sufficient social support in esophageal cancer patients.

### Statistical analysis

Descriptive statistics were used to present demographic and clinical characteristics and the frequency and severity of symptom estimates among esophageal cancer patients. T-tests and one-way ANOVAs were used to examine group differences in demographic and clinical information in QoL. Exploratory factor analysis (EFA) with principal axis factoring was used to identify symptom clusters on the 18 possible symptoms (13 core symptoms and five gastrointestinal cancer-specific symptoms from MDASI-GI) [26]. For the EFA, we examined the Kaiser–Meyer–Olkin (KMO) measure and Bartlett’s test of sphericity. Besides, the components with eigenvalues > 1 were extracted, and factor loadings ≥ 0.5 were considered [27]. After determining the SCs, we calculated a composite score for each SCs. The composite score was calculated as an average distress rating from all the components symptoms from each SCs.

Then, the correlation coefficients (*r*) in continuous variables (SCs, social support, and QoL) were tested using Pearson’s correlation analysis. Multiple regression analysis was employed to analyze which variables might be factors of QoL. Each SCs and social support were the independent variables. The sociodemographic and clinical variables in the univariate analysis (*P* < 0.20) were included in the regression model. SPSS 20.0 software was used to conduct statistical analysis, and the significance level was *P* value < 0.05.

## Results

### Reliability of assessment scales

All scales presented good internal consistency in our study: Cronbach’s α coefficient = 0.919 for FACT-G, 0.954 for MDASI-GI, and 0.917 for MSPSS.

### Characteristics of participants

In the present study, 118 patients effectively completed the survey among 135 cancer patients, and the effective response rate was 87.4%. Seventeen patients were excluded due to invalid data. Finally, 118 esophageal cancer patients were included in our study.

The age of participants ranged from 43 to 85 years old (Mean = 64.84, SD = 8.59). More than 68% were above 60 years old, and 94.1% were male. Among participants, 87.3% reported being married or cohabiting, 67% got a diploma from middle school or below, 74.6% had a monthly income level of ≤ 3000 yuan (RMB), 55.9% were living in rural areas, and 30% had chronic diseases. Of these participants, 18.6% had time after diagnosis above two years, and 61% and 18.1% had received chemotherapy, and surgery, respectively. Details of demographic and clinical information are displayed in Table 1.

**Table 1:** Demographic and clinical characteristics related to the quality of life among esophageal cancer patients.

Variable	Number (%)	Quality of life (FACT-G)			
		Mean	SD	*t*/*F*	*P*
Age				0.917	0.402
< 60	37 (31.4)	68.57	16.07		
60–70	48 (40.7)	68.44	19.47		
≥ 70	33 (28.0)	73.45	17.30		
Gender				1.163	0.247
Male	111 (94.1)	70.36	17.87		
Female	7 (5.9)	62.29	16.80		
Residence				0.728	0.468
Urban	52 (44.1)	71.23	16.98		
Rural	66 (55.9)	68.82	18.56		
Marital status				1.333	0.185
Single or others	103 (87.3)	69.05	17.71		
Married/cohabited	15 (12.7)	75.60	18.37		
Educational level				0.084	0.920
Primary school or under	25 (21.2)	69.04	21.33		
Middle school	54 (45.8)	69.61	18.20		
Senior high school	39 (33.1)	70.79	15.16		
Monthly household income (RMB)				1.432	0.243
< 2000	39 (33.1)	70.21	20.05		
2000–3000	49 (41.5)	67.08	16.00		
> 3000	30 (25.4)	74.03	17.41		
Have chronic diseases				1.691	0.094
No	82 (69.5)	71.71	18.44		
Yes	36 (30.5)	65.72	15.87		
Time since diagnosis				0.065	0.937
< 1 year	76 (64.4)	70.32	17.30		
1–2 year	20 (16.9)	69.30	19.41		
> 2 year	22 (18.6)	68.91	19.04		
Have chemotherapy				1.646	0.102
No	46 (39.0)	66.52	17.81		
Yes	72 (61.0)	72.03	17.66		
Surgery				1.445	0.151
No	96 (81.4)	68.75	17.66		
Yes	22 (18.6)	74.82	18.23		

*SD* Standard deviation, *FACT-G* Functional Assessment of Cancer Therapy-General

### Prevalence and severity of symptoms

Regarding the severity of symptoms, the six most severe symptoms included difficulty in swallowing (Mean = 4.81, SD = 3.64), appetite loss (Mean = 3.51, SD = 3.09), fatigue (Mean = 3.41, SD = 2.74), distress (Mean = 3.23, SD = 2.95), and disturbed sleep (Mean = 3.23, SD = 2.86) and pain (Mean = 3.06, SD = 2.87). The two symptoms with the lowest severity were diarrhea (Mean = 1.90, SD = 2.38) and numbness (Mean = 2.09, SD = 2.55). Regarding the prevalence of symptoms, difficulty in swallowing (83.9%) and fatigue (83.1%) were the two symptoms with the highest occurrence in these participants. In comparison, diarrhea (54.2%) and numbness (58.5%) symptoms had the lowest occurrence. More detailed information is presented in Table 2.

**Table 2:** Severity and prevalence of symptoms among esophageal cancer patients

Symptom	Severity (Mean ± SD)	Prevalence *n* (%)			
		None	Mild	Moderate	Severe
Pain	3.06 ± 2.87	32 (27.1)	39 (33.1)	38 (32.2)	9 (7.6)
Fatigue	3.41 ± 2.74	20 (16.9)	49 (41.5)	38 (32.2)	11 (9.3)
Nausea	2.91 ± 3.04	39 (33.1)	39 (31.1)	27 (22.9)	13 (11.0)
Disturbed sleep	3.23 ± 2.95	29 (24.6)	39 (33.1)	37 (31.4)	13 (11.0)
Distressed	3.23 ± 2.86	25 (21.2)	45 (38.1)	35 (29.7)	13 (11.0)
Shortness of breath	2.26 ± 2.55	45 (38.1)	39 (33.1)	31 (26.3)	3 (2.5)
Memory decline	2.35 ± 2.41	39 (33.1)	48 (40.7)	28 (23.7)	3 (2.5)
Appetite loss	3.51 ± 3.09	28 (23.7)	37 (31.4)	38 (32.2)	15 (12.7)
Sleepy	2.70 ± 2.59	36 (30.5)	38 (32.2)	39 (33.1)	5 (4.2)
Dry mouth	2.86 ± 2.71	34 (28.8)	41 (34.7)	34 (28.8)	9 (7.6)
Sadness	2.74 ± 2.77	41 (34.7)	38 (32.2)	30 (25.4)	9 (7.6)
Vomiting	2.56 ± 3.04	47 (39.8)	36 (30.5)	21 (17.8)	14 (11.9)
Numbness	2.09 ± 2.55	49 (41.5)	40 (33.9)	24 (20.3)	5 (4.2)
Constipation	2.94 ± 2.95	31 (26.3)	48 (40.7)	25 (21.2)	14 (11.9)
Diarrhea	1.90 ± 2.38	54 (45.8)	39 (33.1)	21 (17.8)	4 (3.4)
Difficulty swallowing	4.81 ± 3.64	19 (16.1)	34 (28.8)	26 (22.0)	39 (33.1)
Change in taste	2.58 ± 2.96	42 (35.6)	40 (33.9)	24 (20.3)	12 (10.2)
Abdominal distention	2.40 ± 2.91	45 (38.1)	43 (36.5)	19 (16.1)	11 (9.3)

*SD* Standard deviation

### Symptom clusters

Table 3 shows the composition of symptom clusters. An EFA and a varimax rotation were used to identify the latent constructs of the 18 symptoms in our study. The value of the Kaiser–Meyer–Olkin (KMO) measure was 0.924, and Bartlett’s test of sphericity was < 0.001, indicating that the data were suitable for factor analysis. Four factors were retained (eigenvalue > 1). Therefore, four SCs were identified, accounting for 74% of the total variance. The SCs were labeled (a) “psychological-somatic SC”, which was composed of distress, shortness of breath, memory decline, sleep, dry mouth, sadness, numbness, and abdominal distention; (b) “dysphagia SC”, which was composed of nausea, appetite loss, vomiting, and difficulty swallowing; (c) “fain-pain SC”, which was composed of pain, fatigue, and disturbed sleep; (d) “gastrointestinal SC”, which was composed of constipation, diarrhea, and change in taste.

**Table 3:** Summary of factor analysis of symptoms among esophageal cancer patients

Symptom	Factor loading			
	Factor 1	Factor 2	Factor 3	Factor 4
Distressed	0.622			
Shortness of breath	0.685			
Memory decline	0.729			
Sleepy	0.554			
Dry mouth	0.698			
Sadness	0.691			
Numbness	0.674			
Abdominal distention	0.586			
Nausea		0.663		
Appetite loss		0.577		
Vomiting		0.784		
Difficulty swallowing		0.631		
Pain			0.750	
Fatigue			0.849	
Disturbed sleep			0.579	
Constipation				0.739
Diarrhea				0.840
Change in taste				0.567
Cronbach’s α	0.929	0.864	0.843	0.809
Eigenvalue	4.535	3.258	3.059	2.467
Variance explained	25.195	18.100	16.997	13.708
Total variance explained	25.195	43.295	60.291	73.999

Factor 1, Psychological-somatic symptom cluster; Factor 2, Dysphagia symptom cluster; Factor 3, Fatigue-pain symptom cluster; Factor 4, Gastrointestinal symptom cluster

### Correlations among SCs, social support, and QoL

According to the results of the correlation analysis (Table 4), social support was positively correlated with QoL (*r* = 0.494, *P* < 0.01), while all SCs were negatively associated with QoL (*r *= − 0.446 to − 0.545, *P* < 0.01). Besides, the mean score of QoL (FACT-G) was 69.88 (SD = 17.85) among 118 esophageal cancer patients.

**Table 4:** The correlation analysis between symptom clusters, social support, and quality of life

Variable	FACT-G	Mean	SD
Psychological-somatic symptom cluster	− 0.545*	2.58	2.19
Dysphagia symptom cluster	− 0.509*	3.44	2.71
Fatigue-pain symptom cluster	− 0.447*	3.23	2.49
Gastrointestinal symptom cluster	− 0.467*	2.47	2.36
Social support	0.494*	65.74	11.72
FACT-G	–	69.88	17.85

*SD* Standard deviation, *FACT-G* Functional Assessment of Cancer Therapy-General

*P < 0.01

### Factors of the QoL

The results of the regression analysis for QoL are shown in Table 5. An Independent association was found between psychological-somatic SC (*β *= − 0.299, *P* = 0.023) and QoL. Chemotherapy (*β* = 0.140, *P* < 0.045) and social support (*β* = 0.407, *P* < 0.001) contributed to improving QoL after adjusting demographic and clinical characteristics. Furthermore, the regression model accounted for 47.8% of the variance in QoL among esophageal cancer patients.

**Table 5:** Multiple linear regression analysis of quality of life in esophageal cancer patients

Variable	Quality of life (QoL)				
	*B*	*SE*	*β*	*T*	*P*
Marital status	3.106	3.665	0.058	0.847	0.399
Have chronic diseases	− 1.834	1.643	− 0.081	− 1.117	0.267
Have chemotherapy	5.119	2.524	0.140	2.028	0.045
Have surgery	4.967	3.273	0.109	1.518	0.132
Psychological-somatic SC	− 2.687	1.061	− 0.329	− 2.532	0.013
Dysphagia SC	− 0.632	0.811	− 0.096	− 0.780	0.437
Fatigue-pain SC	− 0.532	0.762	− 0.074	− 0.698	0.487
Gastrointestinal SC	− 0.1153	0.758	− 0.020	− 0.201	0.841
Social support	0.623	0.106	0.409	5.883	< 0.001

Model *F* = 12.922, *P* < 0.001; *R*^2^ = 0.518, Adjusted *R*^2^ = 0.478

*B*, Unstandardized beta, *β*, Standardized regression weight, SE, Standard error, SC, Symptom cluster

## Discussion

Our results showed that most esophageal cancer patients were suffering from multiple symptoms, and four SCs were identified, including psychological-somatic SC, dysphagia SC, fain-pain SC, and gastrointestinal SC. Furthermore, a significant effect on QoL for chemotherapy, psychological-somatic SC, and social support in esophageal cancer patients.

The present study assessed QoL among patients with esophageal cancer in China. Compared to other studies using FACT-G in the USA (Mean = 77.4, SD = 16.3) [28] or Canada (Mean = 80.9, SD = 18) [29] among esophageal cancer patients, lower scores were found in our study (Mean = 69.88, SD = 17.85). This might be because the tumor characteristics and preferred treatment strategies vary between Asian and Western countries [30, 31]. Van den Boorn et al also found differences in QoL between Asian and Western countries[32]. Furthermore, our study’s level of esophageal cancer patients’ QoL was worse than gastric [33] or colorectal cancer [34]. This result indicated that esophageal cancer patients had poor QoL. Therefore, it is urgent to focus on esophageal cancer patients’ QoL and targeted solutions to enhance their QoL.

In the present study, difficulty swallowing and fatigue were esophageal cancer patients’ most common physical symptoms. This result was consistent with previous studies [12]. In the current study, no findings were reported that difficulty in swallowing and fatigue significantly affected esophageal cancer patients’ QoL. However, evidence found that dysphagia weakened cancer patients’ QoL [35]. Most patients experienced progressive dysphagia and rapid weight loss in the early phase of esophageal cancer. Furthermore, esophageal cancer patients with dysphagia reported a feeling of anger, disappointment, lack of confidence, anxiety, and depression, which could negatively affect their QoL [36]. Fatigue is a common physical symptom among cancer patients. Previous studies showed that fatigue was related to inflammation, poor sleep quality, and QoL among patients with advanced cancer [37]. Besides, Wu et al thought that fatigue adversely contributed to esophageal cancer patients’ QoL [38]. In addition to difficulty swallowing and fatigue, Guo et al found that reflux, disturbed sleep, and lack of appetite were the most frequent physical symptoms among esophageal cancer patients [13]. Possible reasons for this phenomenon are that patients received various treatments, and different measurement tools were used to assess symptoms.

Four symptom clusters were determined and named in the current study: psychological-somatic SC (distressed, shortness of breath, memory decline, sleepy, dry mouth, sadness, numbness, and abdominal distention), dysphagia SC (nausea, appetite loss, vomiting, and difficulty swallowing), fain-pain SC (pain, fatigue, and disturbed sleep), and gastrointestinal SC (constipation, diarrhea, and change in taste). Significantly, previous studies identified the pain/fatigue SC, eating difficulties SC, and gastrointestinal SC in patients with esophageal cancer [12], gastrointestinal SC in head and neck cancer (HNC) [39, 40] or esophageal cancer patients [13], and psychological SC in patients with advanced lung cancer [41], but the symptoms in each group were not all the same. For instance, Wikman et al [12] investigated three symptom clusters (fatigue/pain SCs comprising the symptoms of fatigue, esophageal pain, pain, dyspnea, and insomnia; reflux/cough SCs including reflux, coughing, trouble with taste, and dry mouth; eating difficulties SCs comprising eating difficulty, appetite loss, nausea/vomiting, dysphagia, and diarrhea) in post-op esophageal cancer patients using the European Organization for Research and Treatment of Cancer (EORTC) QLQC30 and the esophageal-specific module QLQ-OES18. Li et al [39] classified three symptoms of appetite loss, constipation, and nausea as gastrointestinal SC, the four symptoms of pain, distress, shortness of breath, and sadness as fatigue SC, the two symptoms of dry mouth and mucus into HNC-specific SC, the two symptoms of difficulty swallowing and difficulty with voice and speech into tracheostomy-related SC, the three symptoms of fatigue/weakness, restless and sleepy into fatigue SC, and the seven symptoms of coughing, memory decline, numbness, vomiting, the problem with tasting food, skin pain/burning/rash, and mouth/throat scores into independent of the SC in HNC patients. Symptoms may change at different stages of the disease, which could explain the inconsistencies of SCs. Besides, the latent impact of statistical methods and measurement tools may affect the SCs.

Our results indicated that psychological-somatic SC (distress, shortness of breath, memory decline, sleep, dry mouth, sadness, numbness, and abdominal distention) negatively influenced esophageal cancer patients’ QoL. This SC is less common, and distress and sadness have been considered essential factors of QoL [13, 42, 43]. Dry mouth and appetite loss may be caused by oral mucosal inflammation owing to chemoradiotherapy [44], and abdominal distention and shortness of breath may be associated with postoperative digestive tract reconstruction due to esophageal cancer [44, 45], which therapy would result in severe complications, and decreased QoL in the esophageal cancer patient. Therefore, symptom management programs should be developed to address psychological-somatic symptoms to increase QoL among esophageal cancer patients.

The findings suggested that perceiving more social support contributed to better QoL among esophageal cancer patients. Our results are consistent with previous studies on other cancer patients [18, 46]. Social support, particularly family support, is urgently needed because the family is the bedrock of Chinese society, and cancer patients need to perceive care and concerns from family members. However, esophageal cancer patients might not ask for support from friends or another influential group due to stigma, such as difficulty swallowing or food regurgitation, which may lead to withdrawal from the social situation and isolation [6]. Thus, interventions that increase emotional and social support from family and friends are necessary for esophageal cancer patients.

Besides, our results indicated that esophageal cancer patients receiving chemotherapy had a better QoL than those without chemotherapy, which was consistent with previous findings. Lv et al [47] found that the QoL of esophageal cancer patients with chemoradiotherapy after six months is better than before therapy, and chemoradiotherapy had a lower impact than surgery. Furthermore, other studies thought that SCs would change over time during peri-chemotherapy [48, 49]. For example, Li et al [49] reported that two new SC that appeared after chemotherapy were defined as the chemotherapy SC and the gastrointestinal SC. Therefore, our further research needs to understand the symptom cluster trajectories in patients with esophageal cancer, which may be beneficial in preventing and managing multiple concurrent symptoms.

### Implications

The current study has some practical implications. First, symptom cluster management programs should address dysphagia and fatigue symptoms as they are the most common physical symptoms. For example, esophageal cancer patients should be encouraged to participate in individualized nutritional and habit education to actively prevent dysphagia problems. Besides, nurses and doctors need to pay attention to patients receiving chemoradiotherapy or surgery due to treatment side-effects symptoms, such as dry mouth, appetite loss, and shortness of breath. Second, clinically, some less prominent symptom clusters, including distress, sadness, appetite loss, and dry mouth, are often easily ignored by health managers. However, our study found those to be the independent risk affecting physical and emotional well-being in our study. Finally, more effort should be made to increase social support. Most patients who receive sufficient support from family and friends could positively face multiple sources of stress derived from their diseases. Thus, the family should adequately spend time with patients and provide reassurance.

### Limitations

Several limitations should be considered in the study. First, our study was a cross-sectional study that limited the assessment of the causal relations between variables. Hence, further longitudinal studies are needed to validate our findings, observe the changes and identify more stable SCs. Second, a convenience sample was used in the present study, limiting the results' generalization to other cancer patients. Third, response bias is inevitable because our study used a self-reporting questionnaire to obtain data. Our findings would be overestimated or underestimated by respondents. Finally, the relatively small sample size may influence the significant relationships between variables. Therefore, we need to interpret the results and conclusions of our study carefully.

## Conclusions

In the present study, esophageal cancer patients experienced a relatively low level of QoL. Four SCs were identified: psychological-somatic SC, dysphagia SC, fain-pain SC, and gastrointestinal SC. Besides, psychological-somatic SC negatively affected QoL, while chemotherapy and social support positively affected QoL. Our findings emphasized the importance of psychological-somatic SC management programs and increasing social support to improve QoL among esophageal cancer patients.

## Data Availability

The dataset in this study is available from the corresponding author upon reasonable request.

## Abbreviations

QoL: Quality of life
MSPSS: Multidimensional scale of perceived social support
MDASI-GI: MD Anderson Symptom Inventory Gastrointestinal Cancer Module
PWB: Physical well-being
SFWB: Social/family well-being
EWB: Emotional well-being
FWB: Functional well-being
FACT-G: Functional Assessment of Cancer Therapy-General
SCs: Symptom clusters
